# Host Immunological Effects of Partial Splenic Embolization in Patients with Liver Cirrhosis

**DOI:** 10.1155/2018/1746391

**Published:** 2018-07-15

**Authors:** Yasushi Matsukiyo, Hidenari Nagai, Teppei Matsui, Yoshinori Igarashi

**Affiliations:** Division of Gastroenterology and Hepatology, Department of Internal Medicine (Omori), School of Medicine, Faculty of Medicine, Toho University, 6-11-1 Omorinishi, Ota-ku, Tokyo 143-8541, Japan

## Abstract

**Purpose:**

Restoration of the balance between T lymphocyte subsets and between Th1/Th2 cytokines together with improvement of antitumor immunity has been reported after hepatosplenectomy in patients with liver cirrhosis (LC) and hepatocellular carcinoma (HCC). However, the detailed effects of partial splenic embolization (PSE) on host immunity are unknown. Accordingly, this study evaluated host immunity in patients with cirrhosis receiving PSE for thrombocytopenia.

**Methods:**

Twenty-three adult Japanese patients with cirrhosis and thrombocytopenia underwent PSE using straight coils at our hospital between 2010 and 2015. Blood samples were collected before PSE and 4 weeks after PSE.

**Results:**

The platelet counts were significantly higher 4 weeks after PSE compared with before PSE. The white blood cell count (neutrophils, lymphocytes, and monocytes) also increased significantly after PSE. Furthermore, Th1 cells and Th2 cells showed a significant increase at 4 weeks after PSE compared with before PSE, although there was no significant change of Treg cells. Moreover, serum levels of TNF-alpha, soluble TNF receptor I, and soluble Fas were significantly increased after PSE. There was no significant change of the Child-Pugh score.

**Conclusions:**

In patients with cirrhosis and thrombocytopenia, PSE not only promoted the recovery of leukopenia and thrombocytopenia but also induced activation of host immunity.

## 1. Introduction

Splenectomy is considered to be associated with a potential risk of infection, although it is effective in increasing the platelet count. A meta-analysis of studies involving 19,680 patients who underwent splenectomy demonstrated that the incidence of postsplenectomy sepsis was low among adult patients and a higher mortality rate was only observed among children [1]. Partial splenic embolization (PSE) via catheter intervention is an effective alternative to splenectomy that is less invasive than surgery [2–5]. PSE not only increases the platelet counts but also ameliorates portal hypertension and reduces esophageal varices by decreasing blood flow from the splenic vein [6].

The spleen is the largest lymphoid organ in the body and is important for host defenses against disease-causing organisms. Among multiple roles of the spleen in the immune system, it is critical for T cell function [7–9]. However, splenectomy does not impair T cell function in patients with hepatocellular carcinoma (HCC) and liver cirrhosis but promotes recovery of the balance between T lymphocyte subsets, and Th1/Th2 cytokines improve antitumor immunity [10]. Although some studies have examined differences in immunocompetence after embolization or splenic injury [11–14], it is not clear how PSE influences host immunity in patients with thrombocytopenia. Accordingly, this study was performed to evaluate host immunity in patients with cirrhosis and thrombocytopenia who underwent PSE.

## 2. Methods

### 2.1. Patients

Twenty-three adult Japanese patients with cirrhosis and thrombocytopenia underwent PSE with straight coils at our hospital between 2010 and 2015. Blood samples were collected before PSE and 4 weeks after PSE. Each sample was drawn up into a serum tube and centrifuged at 1800*g* for 10 min to obtain serum that was stored at −80°C. Because the serum level of vascular endothelial growth factor (VEGF) increases over time due to degranulation of platelets [15], samples were processed within 30 min. Serum concentrations of VEGF were measured in duplicate with an enzyme-linked immunosorbent assay (ELISA) kit (Quantikine Human VEGF Immunoassay; R&D Systems, Minneapolis, MN, USA) by an investigator who was blinded to the clinical information about the patients. The serum level of thrombopoietin (TPO) was also measured by ELISA (Quantikine, R&D Systems, Minneapolis, MN, USA). Written informed consent was obtained from each patient after the complications of PSE were fully explained.

### 2.2. Partial Splenic Embolization Procedure

**Figure 1:**
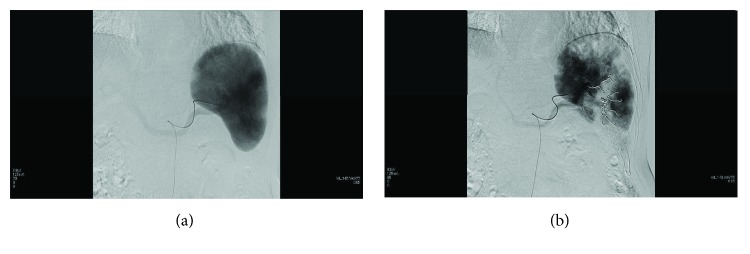
Procedure for partial splenic embolization. The catheter was advanced to the hilum of the splenic artery. Branches of splenic arteries were embolized with microcoils and pieces of gelatin sponge, targeting about 60% embolization.

A catheter was inserted into the right femoral artery under local anesthesia with 1% lidocaine and was advanced until it reached the hilum of the splenic artery. Then, branches of the splenic artery were embolized by using microcoils and pieces of gelatin sponge, with the objective being to achieve about 60% embolization of the spleen [16] (Figure 1).

### 2.3. Analysis of CD4-Positive T Cell Subsets

**Figure 2:**
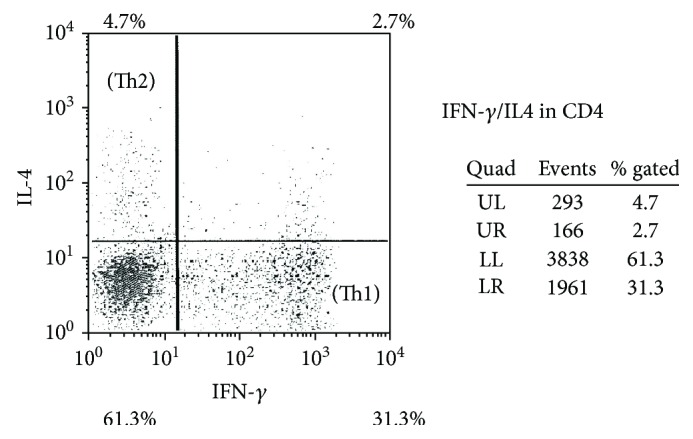
Flow cytometric detection of interferon- (IFN-) *γ* and interleukin- (IL-) 4 in CD4-positive T cells. Upper left: IFN-*γ*-negative/IL-4-positive cells (Th2 cells); lower right: IFN-*γ*-positive/IL-4-negative cells (Th1 cells); upper right: IFN-*γ*/IL-4 double positive cells (Th0 cells).

**Figure 3:**
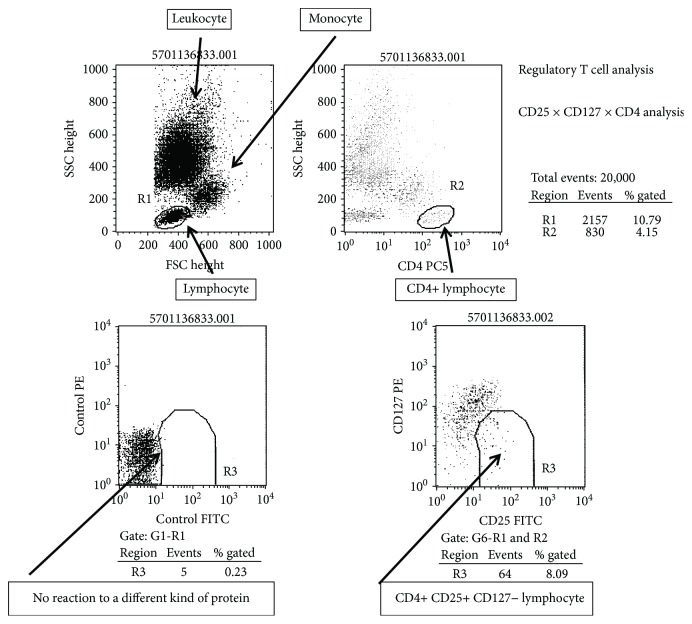
Flow cytometric detection of CD25 FITC and CD127 PE in CD4-positive T cells. Upper left: leukocytes, monocytes, and lymphocytes; upper right: CD4-positive lymphocytes; lower left: no detection of a different protein (control); lower right: CD4-positive/CD127-negative lymphocytes (Treg).

CD4-positive T cell subsets in the peripheral blood were analyzed after nonspecific stimulation with phorbol 12-myristate 13-acetate (PMA), ionomycin, or brefeldin A (Sigma Chemical Co., St. Louis, MO, USA), according to the modified method of Jung et al. [17]. Flow cytometry was used to detect cytoplasmic expression of IFN-gamma and IL-4 by CD4-positive T cells after culture and staining, as reported previously. The percentage of cells producing each cytokine was determined in the total CD4-positive T cell population, which was divided into IFN-gamma-positive/IL-4-negative (Th1) cells and IFN-gamma-negative/IL-4-positive (Th2) cells (Figure 2). In addition, regulatory T cells (Treg cells) were identified as CD25^high^/CD127^low^ cells (Figure 3).

### 2.4. Cytokine Assays

The serum level of tumor necrosis factor-alpha (TNF-alpha) was measured in duplicate by using a commercially available enzyme immunoassay (Quantikine, R&D Systems Inc., Minneapolis, USA). The sensitivity of this assay was 2.6 pg/ml, the intra-assay coefficient of variation was ±8.8%, and the interassay coefficient of variation was ±16.7%. In addition, the serum level of soluble TNF-alpha receptor I (sTNFr-I) was quantified by ELISA (Quantikine, R&D Systems Inc.), and the serum level of soluble Fas (sFas) was measured by a sandwich enzyme immunoassay (Quantikine, R&D Systems Inc.). Moreover, sFas ligand (sFas L) was quantified with an immunoassay kit (MBL, Tokyo, Japan). In this immunoassay, concomitant measurement of seven sFas L standards with known concentrations (0.16, 0.31, 0.63, 1.25, 2.5, 5, and 10 ng/ml) was performed together with patient samples and the detection limit for sFas L was <50 pg/ml. Each assay was performed according to the manufacturer's recommendations [18].

### 2.5. Statistical Analysis

Statistical analysis was done with the Statistical Package for the Social Sciences version 11.0 (SPSS, Chicago, IL, USA). Results are expressed as the mean ± standard deviation (SD). Wilcoxon's signed-rank sum test was used to compare patient characteristics within each group, and a probability of less than 0.05 was considered to indicate statistical significance in all analyses.

This study was approved by the Ethical Review Board of Toho University Medical Center, Omori Hospital (number M17086).

## 3. Results

Twenty-three adult Japanese patients with thrombocytopenia underwent PSE at our hospital between 2010 and 2015. They included 15 men and 8 women aged 37 to 82 years (mean ± SD: 63.2 ± 11 years). One patient had HBV-related LC (B-LC), 14 patients had HCV-related LC (C-LC), 6 patients had non-B-non-C LC, and 2 patients had idiopathic portal hypertension (IPH). Excluding the 2 patients with IPH, the Child-Pugh class was A in 5 patients, B in 13 patients, and C in 3 patients. Twelve patients had HCC, which was stage I in 2 patients, stage III in 2 patients, stage IVA in 6 patients, and stage IVB in 2 patients (Table 1).

### 3.1. Changes of Parameters after PSE

**Figure 4:**
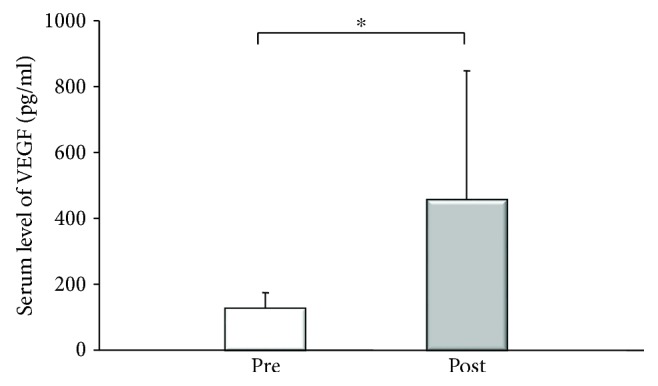
Serum level of vascular endothelial growth factor (VEGF) before and 4 weeks after partial splenic embolization (PSE). Serum VEGF increased significantly after PSE (from 108.8 ± 72 to 426.9 ± 382 pg/ml: ^∗^*p* = 0.0015, by Wilcoxon's test).

**Figure 5:**
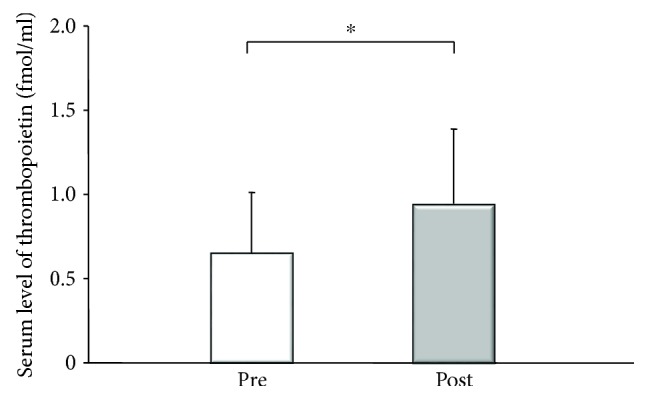
Serum level of thrombopoietin (TPO) before and 4 weeks after partial splenic embolization (PSE). TPO increased after PSE (from 0.69 ± 0.3 to 0.86 ± 0.5 fmol/min: ^∗^*p* = 0.0464, by Wilcoxon's test).

Table 2 lists various parameters before PSE and 4 weeks after PSE. The white blood cell (WBC) count showed a significant increase after PSE compared to before PSE, with elevation of the neutrophil, lymphocyte, and monocyte counts. The platelet count also increased significantly after PSE. Furthermore, serum levels of VEGF (from 108.8 ± 72 to 426.9 ± 382 pg/ml: *p* = 0.0015, by Wilcoxon's test) and TPO (from 0.69 ± 0.3 to 0.86 ± 0.5 fmol/min: *p* = 0.0464, by Wilcoxon's test) increased after PSE (Figures 4 and 5). Similar changes of these parameters were confirmed in the 21 patients without IPH (data not shown). Liver function (Child-Pugh score) did not improve after PSE, although the prothrombin time increased after embolization.

### 3.2. Changes of Peripheral Blood Th1, Th2, and Treg Cells

**Figure 6:**
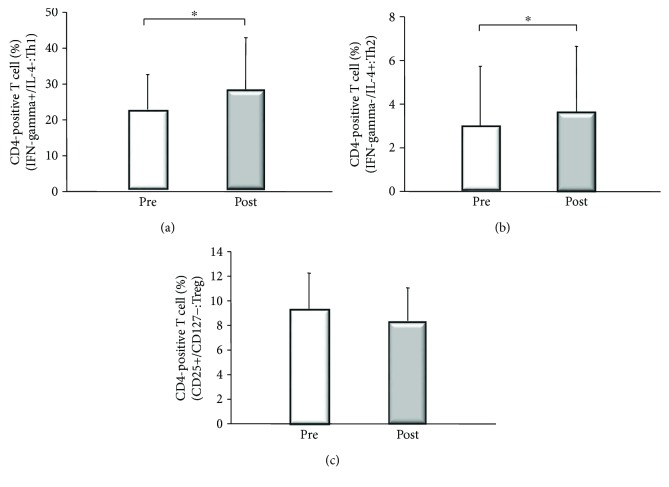
Peripheral blood Th1, Th2, and Treg cells before and 4 weeks after partial splenic embolization (PSE). Upper left: IFN-*γ*-positive/IL-4-negative cells (Th1 cells) showed a significant increased after PSE compared to before PSE (from 22.5 ± 9% to 27.7 ± 12%: ^∗^*p* = 0.0027, by Wilcoxon's test). Upper right: IFN-*γ*-negative/IL-4-positive cells (Th2 cells) also increased significantly after PSE (from 3.2 ± 3% to 3.8 ± 3%: ^∗^*p* = 0.0202, by Wilcoxon's test). Lower: there was no significant change of regulatory T cells (Treg cells), which were identified as CD25^high^/CD127^low^ cells, between before and after PSE (from 9.2 ± 3% to 8.1 ± 2%).

Both Th1 cells and Th2 cells showed significant increase after PSE compared to before PSE (Th1 cells: from 22.5 ± 9% to 27.7 ± 12%; Th2 cells: from 3.2 ± 3% to 3.8 ± 3%) (^∗^*p* = 0.0027 and ^∗^*p* = 0.0202, respectively, by Wilcoxon's test), although there was no significant changes of Treg cells after PSE (from 9.2 ± 3% to 8.1 ± 2%) (Figure 6). These changes of Th1, Th2, and Treg cells were also confirmed after excluding the patients with IPH (data not shown).

### 3.3. Changes of Cytokines

**Figure 7:**
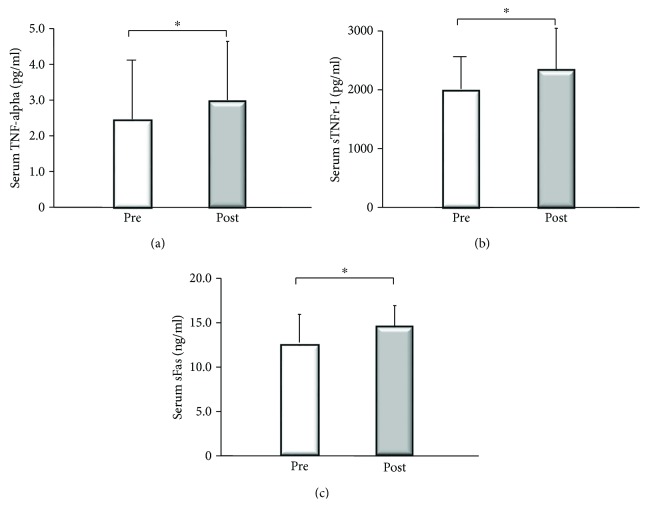
Serum cytokine levels before and 4 weeks after partial splenic embolization (PSE). Upper left: the serum level of TNF-alpha showed a significant increase after PSE compared to before PSE (from 2.4 ± 2 pg/ml to 3.0 ± 2 pg/ml: ^∗^*p* = 0.0077, by Wilcoxon's test). Upper right: the serum level of soluble TNF-alpha receptor I was also increased significantly after PSE compared to before PSE (from 1997.3 ± 603 pg/ml (sTNFr-I) to 2392.0 ± 701 pg/ml: ^∗^*p* = 0.0022, by Wilcoxon's test). Furthermore, the serum level of soluble Fas was significantly higher after PSE than before PSE (from 11.8 ± 3 ng/ml to 13.7 ± 3 ng/ml: ^∗^*p* = 0.0076, by Wilcoxon's test).

The serum levels of TNF-alpha showed significant increase after PSE (from 2.4 ± 2 pg/ml to 3.0 ± 2 pg/ml: ^∗^*p* = 0.0077, by Wilcoxon's test) compared to before PSE, as did the serum level of sTNFr-I (from 1997.3 ± 603 pg/ml to 2392.0 ± 701 pg/ml: ^∗^*p* = 0.0022, by Wilcoxon's test). Serum sFas was also significantly increased after PSE compared to before PSE (from 11.8 ± 3 ng/ml to 13.7 ± 3 ng/ml: ^∗^*p* = 0.0076, by Wilcoxon's test) (Figure 7). The serum levels of sFas L never exceeded 0.15 ng/ml (data not shown). These changes of cytokines were confirmed after excluding the patients with IPH (data not shown).

## 4. Discussion

In patients with cirrhosis and thrombocytopenia undergoing PSE, this study demonstrated significant elevation of the WBC count after embolization compared to before embolization that reflected an increase in neutrophils, lymphocytes, and monocytes. It is possible that the WBC count increased due to release of white cells from the spleen. Bone marrow hyperplasia was reported in hypertensive patients with cirrhosis and hypersplenism [19], while serum levels of M-CSF and GM-CSF were significantly reduced by subtotal splenectomy in cirrhosis patients with splenomegaly secondary to portal hypertension [20]. In our patients with cirrhosis and thrombocytopenia who underwent PSE, the WBC count might not only have been increased by release of white cells from spleen but also by normalization of serum M-CSF and GM-CSF levels with suppression of marrow hyperplasia. After investigating the long-term effects of splenectomy in patients with HCV-related LC, Inagaki et al. reported that liver function was likely to be better 5 years after surgery [21]. We did not find improvement of liver function following PSE (evaluated from the Child-Pugh score), although the prothrombin time increased, but a longer observation period may have been needed because our study only assessed change 4 weeks after embolization. We did not assess liver function at 1 year after PSE in the present study, because some of the patients had HCC and received chemotherapy.

We found that the platelet count was significantly higher after PSE, and serum levels of VEGF and TPO increased as well. Splenic pooling of platelets has been shown to cause thrombocytopenia in patients with splenomegaly [22], and the platelet count might have increased due to release of platelets after embolization in our patients. It was reported that serum TPO increases after splenectomy in patients with chronic immune thrombocytopenic purpura [23], and we confirmed that also TPO increases after PSE. TPO is a physiological regulator of megakaryothrombopoiesis [24], and TPO mRNA expression has been detected in the liver and kidneys of humans [25]. Our findings suggested that the evaluation of the platelet count was also induced by increased hepatic production of TPO after PSE. Butthep et al. found the elevation of VEGF in patients with thalassemia after splenectomy [26]. They reported that hypoxia-inducible factor- (HIF-) alpha was a useful biomarker of cellular hypoxia and was correlated with VEGF in patients with thalassemia [27] and that HIF induced transcription of genes ameliorating the effects of hypoxia, including VEGF [28]. These reports support our detection of an increased serum VEGF level at 4 weeks after PSE.

It was reported that Th1 cytokines suppress liver fibrosis, with interferon-*γ* being a potent inhibitor of the activation of hepatic stellate cells [29, 30], while Th2 cytokines such as IL-4 and IL-3 promote activation of hepatic stellate cells and progression of liver fibrosis [31–33]. Tanabe et al. reported that splenectomy altered the balance of hepatic Th1/Th2 cytokine expression in the direction of Th1 dominance in models of CCl_4_-induced and TAA-induced liver fibrosis [34]. In our study, Th1 cells and Th2 cells showed a significant increase after PSE in patients with cirrhosis, with serum levels of TNF-alpha, sTNFr-I, and sFas also increasing significantly. Fas is an important member of a family of receptors that transduce apoptotic signals leading to programmed cell death. It belongs to the tumor necrosis factor (TNF) receptor superfamily, which includes TNF receptor I (TNFr-I) and TNF receptor II (TNFr-II), which were the first members to be discovered and characterized [35, 36]. These receptors are expressed on the surface of various cells, while their soluble forms are released into the circulation after cleavage of the extracytoplasmic domains or alternate splicing [37]. While the serum level of TNF-alpha was increased after PSE in the present study, serum levels of sTNFr-I and sFas were also increased. Thus, the Th1/Th2 balance was not recognized as altered because both Th1 and Th2 increased, but PSE might have improved liver function by inhibiting fibrosis. Moreover, these results indicate that PSE did not cause severe liver injury since the more abundant TNF-alpha or Fas ligand did not bind to TNF-related apoptosis-inducing ligand or Fas. Th1 and Th2 cells cross-regulate their own development. It has been reported that Th2 cytokines inhibit antitumor immunity [38], while activation of Th1 cytokines promotes antitumor immunity [39–42]. We have previously reported that Th1 dominance is lost in HCC patients due to an increase in Th2 cells and that carcinogenesis might be more likely to occur in patients with chronic HCV infection if they show an increase in Th2 cells [43]. Although elevation of Th1 cells after PSE might upregulate antitumor immunity in LC patients with HCC, further investigation will be needed to confirm this. There are two distinct subsets of Treg cells in the peripheral lymphoid organs, which are natural Treg (nTreg) cells that develop in the thymus after recognition of high-affinity autoantigens and induced Treg (iTreg) cells that develop from conventional T cells following peripheral exposure to antigens and cytokines such as TGF-*β* or IL-10 [44]. These Treg subsets may have synergistic actions or may have different targets that maintain immune homeostasis, although they possibly even have a developmental role [45].

## 5. Conclusion

In patients with cirrhosis and thrombocytopenia, PSE not only led to improvement of leukopenia and thrombocytopenia but also induced activation of host immunity, although further investigation is needed to confirm these findings.

## Figures and Tables

**Table 1:** Clinical characteristics for 23 patients.

Number of patients	23
Mean age	63.2 + 11
Gender (M/F)	15/8
Etiology (HBV/HCV/non-B-non-C/IPH)	1/14/6/2
Child-Pugh classification (A/B/C)	5/13/3
With or without HCC	12/9
Stage of HCC (I/II/III/IVA/IVB)	2/0/2/6/2

HCC: hepatocellular carcinoma.

**Table 2:** Changes of various parameters before and after 4 weeks of PSE.

	Pre-PSE	Post-PSE	*p*
Ammonia (mg/dl)	82.1 ± 94	51.0 ± 43	*0.1140*
Total bilirubin (g/dl)	1.5 ± 0.9	1.6 ± 1.2	*0.6130*
Direct bilirubin (g/dl)	0.6 ± 0.5	0.7 ± 0.9	*0.2650*
Albumin (mg/dl)	3.2 ± 0.6	3.1 ± 0.6	*0.1050*
AST (IU/l)	52.1 ± 30	58.3 ± 44	*0.3890*
ALT (IU/l)	34.8 ± 27	32.5 ± 28	*0.0890*
Total cholesterol (mg/dl)	130.2 ± 30	127.4 ± 21	*0.9650*
Blood urea nitrogen (mg/dl)	16.6 ± 8	15.0 ± 6	*0.1050*
Creatinine (mg/dl)	0.8 ± 0.3	0.8 ± 0.2	*0.1300*
Prothrombin time (%)	65.8 ± 14	69.7 ± 13	***0.013*** ^∗^
White blood cell (/mm^3^)	2945.4 ± 1471	4704.6 ± 1730	***0.001*** ^∗^
Segment (/mm^3^)	1791.2 ± 954	2809.3 ± 912	***0.009*** ^∗^
Lymphocyte (/mm^3^)	607.2 ± 215	1038.0 ± 451	***0.006*** ^∗^
Monocyte (/mm^3^)	245.9 ± 70	440.9 ± 103	***0.001*** ^∗^
Platelets (×10^4^/mm^3^)	4.32 ± 1.5	8.95 ± 5.0	***0.001*** ^∗^
AFP (ng/ml)	2561.3 ± 5704	19806.0 ± 39533	*0.0679*
AFP-L3 (%)	19.3 ± 22	26.2 ± 31	*0.1088*
DCP (AU/ml)	325.2 ± 677	364.5 ± 490	*1.0000*
Child-Pugh score	7.6 ± 1.9	7.4 ± 1.9	*0.4174*

PSE: partial splenic embolization; AST: aspartate aminotransferase; ALT: alanine aminotransferase; AFP: alpha-fetoprotein; DCP: des-gamma carboxyprothrombin. ^∗^Statistically different compared with Wilcoxon's signed-rank sum test.
